# Gender and time delays in diagnosis of pulmonary tuberculosis: a cross-sectional study from China

**DOI:** 10.1017/S0950268819000049

**Published:** 2019-02-22

**Authors:** H. G. Chen, T. W. Wang, Q. X. Cheng

**Affiliations:** 1 Peking University Institute of Mental Health, National Clinical Research Center for Mental Disorders (Peking University Sixth Hospital), Key Laboratory of Mental Health, Ministry of Health (Peking University), Beijing, 100191, China; 2 Yulin Center for Disease Control and Prevention, Shaanxi, 719000, China

**Keywords:** Community epidemics, infectious disease epidemiology, meningitis – tuberculous, public health

## Abstract

Gender inequality has severe consequences on public health in terms of delay in diagnosis of pulmonary tuberculosis (PTB). In order to explore gender-related differences in diagnosis delay, a cross-sectional study of 10 686 patients diagnosed with PTB in Yulin from 1 January 2009 to 31 December 2014 was conducted. Diagnosis delay was categorised into ‘short delay’ and ‘long delay’ by four commonly used cut-off points of 14, 30, 60 and 90 days. Logistic regression analysis was used to analyse gender differences in diagnostic delay. Stratified analyses by smear results, age, urban/rural were performed to examine whether the effect persisted across the strata. The median delay was 31 days (interquartile range 13–65). Diagnostic delay in females at cut-off points of 14, 30, 60 and 90 days had odds ratios (OR) of 0.99 (95% CI 0.91–1.09), 1.09 (95% CI 1.01–1.18), 1.15 (95% CI 1.05–1.26) and 1.18 (95% CI 1.06–1.31), respectively, compared with males. Stratified analysis showed that females were associated with increased risk of longer delay among those aged 30–60 years, smear positive and living in the rural areas (*P* < 0.05). The female-to-male OR increased along with increased delay time. Further inquiry into the underlying reasons for gender differences should be urgently addressed to improve the current situation.

## Introduction

Although progress has been made in global pulmonary tuberculosis (PTB) control, the disease remains a major portion of the global disease burden and one of the most intractable health challenges in low- and middle-income countries [1, 2]. Over the past years, a marked increase in the number of patients diagnosed with PTB was reported [3–5]. China contributed a significant portion to this increase. The results of the Fifth National Epidemiological Survey on PTB in China indicated that the prevalence of PTB at the national level *demonstrated a slow downward trend* [6]. It will be difficult to achieve the goal of ending the epidemic of PTB by 2035 given the current trend [7, 8]. Early diagnosis and adequate treatment are the essential components for reducing the incidence of PTB and *achieving goals of the End TB Strategy* [9]. It is known that the delay in diagnosis of PTB leads to prolonged spread of PTB, increases severity of the disease and results in more extensive diseases and complications [6, 10–13]. In many developing countries today, females undertake a double or triple workload, i.e. taking care of the family and home, doing agricultural work and perhaps also doing waged work. The impact of tuberculosis in females is thus severe not only on their families but also on the development of society through the loss of workforce, ruined families and orphaned children [14–16]. Therefore, it is urgent to explore the association between gender and diagnosis delay of PTB. To address this knowledge gap, the World Health Organization (WHO) has already proposed a framework and priorities for research on gender and TB [17].

In China, few studies had focused on gender-related differences in diagnosis delay. Most studies were exploring the potential risk factors for diagnosis delay [6, 18, 19]. In these studies, gender was mostly defined as one of the confounding factors and not an important influencing factor. Moreover, most of the available studies involving gender used different definitions of diagnosis delays as well as varying classifications of socio-demographic variables. Accordingly, the results varied and failed to provide consistent and objectively quantitative evidence. These may explain why the impact of gender disparity has not been given enough attention and concern. This study was designed to evaluate the gender-related differences in diagnosis delay defined by four commonly used classification criteria worldwide under age-specific, smear-specific and urban/rural-specific conditions.

## Methods

**Fig. 1.**
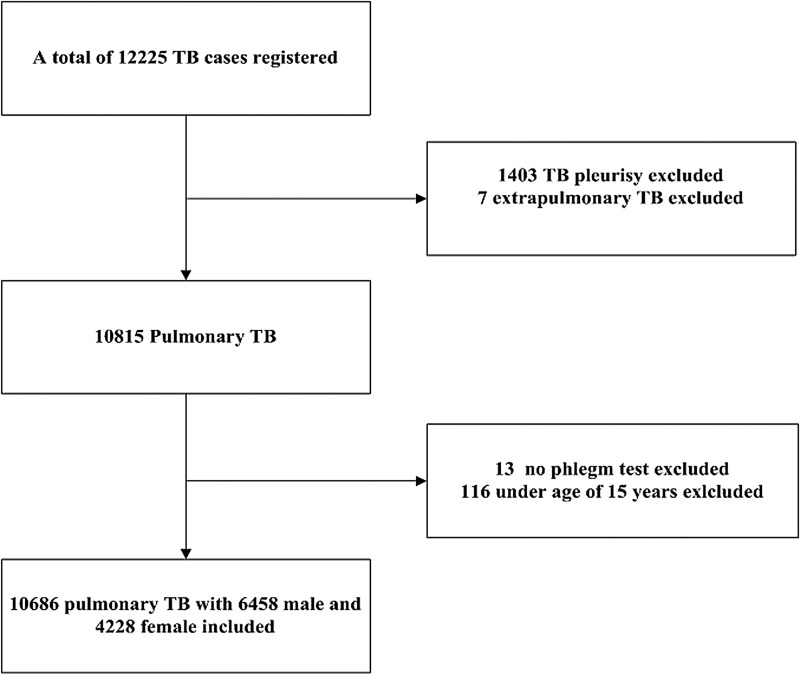
Flow diagram for selection of 10 686 subjects in Yulin city from 1 January 2009 to 31 December 2014.

An institution-based cross-sectional study was conducted among all PTB patients diagnosed from 1 January 2009 to 31 December 2014 in all 12 PTB dispensaries located in Yulin. A total of 10 686 qualified PTB cases were included in the study after excluding patients under age 15, patients without sputum test as well as patients diagnosed as pleurisy and extra pulmonary TB. Detailed flow diagram for subject selection is shown in Figure 1.

The recommended standard operating procedures for the diagnosis of PTB were to collect and examine a total of three sputum specimens from suspected patients with respiratory symptoms in two consecutive days. Microscopic examination of sputum for the presence of acid-fast bacilli was performed at PTB dispensaries.

Informed consent forms were obtained from all subjects.

A semi-structured questionnaire was administered to collect the intended data by trained doctors and health care workers before the initiation of PTB treatment. Parameters such as gender, age, household registration status (local and migrant) through the Chinese Hukou system, occupations, sources of PTB, smear test results and rural/urban residents were included in the questionnaire which were part of the surveillance. Ethical clearance was obtained from medical research coordinating committee of Yulin CDC.

Date of onset of PTB symptoms was defined as the day the patient remembered feeling sick for the first time with at least one active PTB symptom. The date of the PTB patient's first visit to a health care facility (health centres or hospitals) was recorded in the questionnaire.

Diagnosis delay was defined as the duration from onset of PTB symptoms noted by the patient to the date of the first visit to a health care facility.

In order to comprehensively compare the gender-related difference in diagnosis delay, four delay time groups were defined: group A with 14 days (⩽14 *vs*. >14), group B with 30 days (⩽30 *vs*. >30), group C with 60 days (⩽60 *vs*. >60) and group D with 90 days (⩽90 *vs*. >90).

### Data analysis and statistics

Four cut-off points were used to compare the gender-related diagnosis delay. Pearson's *χ*^2^ test was used to compare the proportions of gender and other characteristics among four groups. Univariate and multivariable logistic regression models were used to calculate odds ratios (ORs) for gender-related diagnosis delay among four categorised groups. Age, occupation, patient source, sputum smear results, household registration status and urban/rural were all included as independent variables in the multivariable logistic regression models. A stratified multivariable logistic regression analysis by smear results, age, urban/rural was conducted to examine whether the effect persisted across the strata. A two-sided *P* value <0.05 was considered significant for all analyses. The database was constructed with EpiData v. 3.1 (EpiData Association, Denmark) and data were analysed using SPSS v. (SPSS Inc., Chicago, IL, USA).

## Results

### Characteristics of subjects among four delay groups

A total of 10 686 patients with PTB were included in the study, including 6458 males (60.4%) and 4228 females (39.6%). The median age was 38 (interquartile range (IQR) 22–60). There were 9153 rural patients accounting for 85.7% of all cases. The median diagnosis delay time for all cases was 31 days (IQR 13–65 days). The median diagnosis delay was 32 days (IQR 13–67) for females and 31 days for males (IQR 14–63) (*P* = 0.110). Among the four groups, a higher proportion of PTB with longer delay was observed in females than in males except group A (see Table 1). Age-related trends were consistent across the four groups with a higher proportion of longer delay among younger (15–30 years) and elder cases (⩾60 years). In addition to age, household registration status, occupation, PTB source, smear results and urban/rural residents were also found to be associated with longer delay in group A. Factors associated with longer delay varied across groups B, C and D.

**Table 1.** Gender and other characteristics among four delay groups in 10 686 cases in Yulin city from 1 January 2009 to 31 December 2014

Variables	Group A (⩽14 *vs*.＞14)		*P*	Group B (⩽30 *vs*. ＞30)		*P*	Group C (⩽60 *vs*. ＞60)		*P*	Group D (⩽90 *vs*. ＞90)		*P*
	*N* (%)	*N* (%)		*N* (%)	*N* (%)		*N* (%)	*N* (%)		*N* (%)	*N* (%)	
Gender												
Male	1741 (27.0)	4717 (73.0)		3177 (49.2)	3281 (50.8)		6458 (78.6)	1758 (21.4)		5425 (84.0)	1033 (16.0)	
Female	1142 (27.0)	3086 (73.0)	0.953	1985 (46.9)	2243 (53.1)	0.023	2970 (70.2)	1258 (29.8)	0.004	3463 (81.9)	765 (18.1)	0.005
Age												
15–30	1402 (30.6)	3178 (69.4)		2386 (52.1)	2194 (47.9)		3472 (75.8)	1108 (24.2)		3988 (87.1)	592 (12.9)	
30–45	382 (26.4)	1063 (73.6)		664 (46.0)	781 (54.0)		1034 (71.6)	411 (28.4)		1217 (84.2)	228 (15.8)	
45–60	436 (23.3)	1439 (76.7)		828 (44.2)	1047 (55.8)		1269 (67.7)	606 (32.3)		1493 (79.6)	382 (20.4)	
⩾60	663 (23.8)	2123 (76.2)	<0.001	1284 (46.1)	1502 (53.9)	<0.001	1895 (68.0)	891 (32.0)	<0.001	2190 (78.6)	596 (21.4)	<0.001
Household												
Local	2794 (26.8)	7642 (73.2)		5026 (48.2)	5410 (51.8)		7490 (71.8)	2946 (28.2)		8672 (83.1)	1764 (16.9)	
Migrant^a^	89 (35.6)	161 (64.4)	0.002	136 (54.4)	114 (45.6)	0.051	180 (72.0)	70 (28.0)	0.937	216 (86.4)	34 (13.6)	0.168
Occupation												
Farmer	1716 (25.1)	5123 (74.9)		3225 (47.2)	3614 (52.8)		4838 (70.7)	2001 (29.3)		5636 (82.4)	1205 (17.6)	
Student	503 (35.4)	917 (64.6)		785 (55.3)	635 (44.7)		1118 (78.7)	302 (21.3)		1254 (88.3)	166 (11.7)	
Service industry^b^	204 (22.5)	703 (77.5)		377 (41.6)	530 (58.4)		612 (67.5)	295 (32.5)		717 (79.1)	190 (20.9)	
Others	460 (30.3)	1060 (69.7)	<0.001	775 (51.0)	745 (49.0)	<0.001	1102 (72.5)	418 (27.5)	<0.001	1283 (84.4)	237 (15.6)	<0.001
PTB source												
Passive	1107 (24.0)	3501 (76.0)		2112 (45.8)	2496 (54.2)		3113 (67.6)	1495 (32.4)		3698 (80.3)	910 (19.7)	
Referral	796 (30.9)	1783 (69.1)		1313 (50.9)	1266 (49.1)		1976 (76.6)	603 (23.4)		2252 (87.3)	327 (12.7)	
Tracking	721 (24.7)	2195 (75.3)		1372 (47.1)	1544 (52.9)		2135 (73.2)	781 (26.8)		2449 (84.0)	467 (16.0)	
Others	259 (44.4)	324 (55.6)	<0.001	365 (62.6)	218 (37.4)	<0.001	446 (76.5)	137 (23.5)	<0.001	489 (83.9)	94 (16.1)	<0.001
Smear results												
Negative	2035 (27.8)	5293 (72.2)		3576 (48.8)	3752 (51.2)		5344 (72.9)	1984 (27.1)		6167 (84.2)	1161 (15.8)	
Positive	848 (25.3)	2510 (74.7)	0.007	1586 (47.2)	1772 (52.8)	0.132	2326 (69.3)	1032 (30.7)	<0.001	2721 (81)	637 (19)	<0.001
Urban/rural												
Urban	509 (33.2)	1024 (66.8)		732 (47.7)	801 (52.3)		1137 (74.2)	396 (25.8)		1347 (87.9)	186 (12.1)	
Rural	2374 (25.9)	6779 (74.1)	<0.001	4430 (48.4)	4723 (51.6)	0.637	6533 (71.4)	2620 (28.6)	0.025	7541 (82.4)	1612 (17.6)	<0.001

a Migrants in China are commonly members of a floating population, which refers primarily to migrants in China without local household registration status through the Chinese Hukou system.

b The service industry involve the provision of general services to other businesses as well as final consumers.

### Association between gender and diagnosis delays

Female gender was associated with longer diagnosis delay among group B (OR, 95% CI 1.09 (1.01–1.18)), group C (OR, 95% CI 1.15 (1.05–1.26)) and group D (OR, 95% CI 1.18 (1.06–1.31)) in multivariable analysis (see Table 2). However, no gender-related diagnosis delay was found in group A with a cut-off point of 14 days. The longer the cut-off point for the delay was, the stronger the association between female and diagnosis delay was shown.

**Table 2.** Association between gender and diagnosis delays with four cut-off points examined in multivariable regression analysis in 10 686 cases in Yulin city from 1 January 2009 to 31 December 2014

Delay groups	Unadjusted OR (95% CI)	Adjusted OR (95% CI) ^^a^^
Group A (<14 *vs*. ⩾14)		
Male	1	1
Female	1.00 (0.91–1.09)	0.99 (0.91–1.09)
Group B (<30 *vs*. ⩾30)		
Male	1	1
Female	1.09 (1.01–1.18)	1.09 (1.01–1.18)
Group C (<60 *vs*. ⩾60)		
Male	1	1
Female	1.13 (1.04–1.23)	1.15 (1.05–1.26)
Group D (<90 *vs*. ⩾90)		
Male	1	1
Female	1.16 (1.05–1.29)	1.18 (1.06–1.31)

a Adjusted for age, household register, occupation, patient source, sputum smear results and urban/rural.

OR, odds ratio; 95% CI, 95% confidence interval.

### Subgroup analysis stratified by age, smear results and urban/rural

Subgroup analysis stratified by age showed that female gender was associated with longer delay in age range 45–59 years in group B (OR, 95% CI 1.24 (1.01–1.51), see Table 3). The associations between gender and longer delay were statistically significant in individual age 30–44 and 45–59 years (*P*<0.05) in groups C and D. Subgroup analysis results stratified by sputum smear results showed that gender was not associated with longer delay across the strata in groups A and B in this study (*P* > 0.05, see Table 4). Female gender showed significant association with longer delay (*P*<0.05) in groups C and D. Subgroup analysis results stratified by urban/rural showed that significant differences were found between gender and diagnosis delay across the strata in groups B, C and D (see Table 5).

**Table 3.** Stratified analysis by age of association between gender and diagnosis delay in 10 686 cases in Yulin city from 1 January 2009 to 31 December 2014

Delay groups	Adjusted OR (95% CI)^a^ among age <30	Adjusted OR (95% CI)^a^ among age ⩾30 and <45	Adjusted OR (95% CI)^a^ among age ⩾45 and <60	Adjusted OR (95% CI)^a^ among age ⩾60
Group A (<14 *vs*. ⩾14)				
Male	1	1	1	1
Female	0.91 (0.80–1.04)	1.06 (0.83–1.35)	1.04 (0.83–1.32)	1.09 (0.90–1.31)
Group B (<30 *vs*. ⩾30)				
Male	1	1	1	1
Female	1.01 (0.89–1.13)	1.16 (0.93–1.44)	1.24 (1.01–1.51)	1.08 (0.92–1.27)
Group C (<60 *vs*. ⩾60)				
Male	1	1	1	1
Female	1.09 (0.95–1.26)	1.44 (1.14–1.83)	1.31 (1.07–1.61)	0.94 (0.79–1.11)
Group D (<90 *vs*. ⩾90)				
Male	1	1	1	1
Female	1.10 (0.92–1.31)	1.50 (1.12–2.03)	1.41 (1.10–1.78)	0.93 (0.76–1.13)

a Adjusted for household register, occupation, patient source, sputum smear results and urban/rural.

OR, odds ratio; 95% CI, 95% confidence interval.

**Table 4.** Stratified analysis by sputum smear results of association between gender and diagnosis delay in 10 686 cases in Yulin city from 1 January 2009 to 31 December 2014

Delay groups	Adjusted OR (95% CI)^a^ among smear- positive cases	Adjusted OR (95% CI)^a^ among smear- negative cases
Group A (<14 *vs*. ⩾14)		
Male	1	1
Female	0.95 (0.81–1.13)	1.01 (0.90–1.12)
Group B (<30 *vs*. ⩾30)		
Male	1	1
Female	1.08 (0.94–1.25)	1.09 (0.99–1.20)
Group C (<60 *vs*. ⩾60)		
Male	1	1
Female	1.19 (1.02–1.38)	1.13 (1.01–1.25)
Group D (<90 *vs*. ⩾90)		
Male	1	1
Female	1.23 (1.03–1.48)	1.14 (1.00–1.30)

a Adjusted for age, household register, occupation, patient source and urban/rural.

OR, odds ratio; 95% CI, 95% confidence interval.

**Table 5.** Stratified analysis by urban/rural of association between gender and diagnosis delay in 10 686 cases in Yulin city from 1 January 2009 to 31 December 2014

Delay groups	Adjusted OR (95% CI)^a^ among urban cases	Adjusted OR (95% CI)^a^ among rural cases
Group A (<14 *vs*. ⩾14)		
Male	1	1
Female	1.03 (0.82–1.30)	0.99 (0.90–1.10)
Group B (<30 *vs*. ⩾30)		
Male	1	1
Female	0.97.98 (0.79–1.20)	1.11 (1.02–1.21)
Group C (<60 *vs.* ⩾60)		
Male	1	1
Female	1.04 (0.82–1.33)	1.17 (1.06–1.28)
Group D (<90 *vs*. ⩾90)		
Male	1	1
Female	0.82 (0.59–1.14)	1.23 (1.10–1.38)

a Adjusted for age, household register, occupation, patient source and sputum smear results.

OR, odds ratio; 95% CI, 95% confidence interval.

## Discussion

This study offered quantitative evidence for gender-related difference in diagnosis delay in PTB patients. Consistent with many studies conducted in diverse settings [6, 10, 20, 21], this study found that the females suffered from longer diagnosis delay than males. However, a substantial number of studies made the opposite conclusion [22, 23]. This might be related to the different cultural factors and health systems between China and elsewhere.

This study indicated that the associations between females and patient delay presented with an enhanced relevance along with increased delay time in diagnosis. This finding was worrying, as previous studies concluded that the adverse consequences of a longer delay at any stage of case detection, diagnosis and treatment might be fatal for patients, and it would also intensify infectivity within the family and community. No gender-related difference in diagnosis delay was found in group A, which indicated that associations between gender and diagnosis delay would be neglected if the cut-off point for the delay was set to only 14 days. Factors for longer delay included individual and provider/system levels. Reported individual-level barriers included physical, financial, stigma, health literacy and socio-cultural factors such as living habits and religious belief [24]. Policymakers should formulate and prioritise gender-specific interventions to improve the condition.

Increased delay risk for female PTB was found among those aged 30–45. Attention should be paid to this disparity for a majority of females of this age group might be young mothers or pregnant. The delay in their visits might cause a huge impact on their children and families and was also a great public concern for PTB prevention and control [12, 15, 25]. The intensity of association was greater among smear-positive cases and increased along with the increase in delay time. Detection, treatment and management of smear-positive patients were recognised as the key points for PTB control, implying that more attention should be paid to female PTB patients. Unlike other studies [26, 27], no gender-related differences were found in urban cases. Those studies were performed in other continents and therefore may be of limited relevance. Rural females were found to be associated with longer delay. Moreover, the delay time and intensity of association showed a trend. This finding was consistent with previous studies performed in rural setting [28–30]. In rural China, females are responsible for agricultural work and household work, including the care of children, which mean that they might have less time than male to seek prompt health care. In addition, rural female patients had lower level of education than males. It was likely that less educated patients would have less access to knowledge about PTB and therefore be unaware of the underlying symptoms of tuberculosis.

Our study, however, has its own limitations. First, the definition of the onset of PTB symptom was subjective. As the self-reporting method used in data collection relied on personal recall, this may have led to recall bias. Diagnosis delay might be underestimated. Nevertheless, there was little reason to suspect the major findings. Since no evidence reflected that the underestimation affected either the female or male gender preferentially. Second, the findings of this study may not be generalisable in different settings because delay diagnosis might be associated with different socio-cultural factors. Additionally, this study had a limited scope to assess the impact of more extensive socio-economic and cultural factors on patient delays, such as per capita income.

This study provided a complementary understanding of gender-related differences in diagnosis delay and critical insights to inform effective gender-specific interventions. Further inquiry into the underlying reasons for gender differences would be beneficial for developing effective policies and measures to improve the current situation. Screening for TB infection among high-risk female populations should be considered as a high priority.
